# Ileocecal valve preservation in a 58-year-old Crohn’s disease patient

**DOI:** 10.1007/s00384-016-2505-x

**Published:** 2016-02-01

**Authors:** Erica P. Turse, Christopher J. Lahr, Nitin K. Gupta

**Affiliations:** Department of Internal Medicine, University of Mississippi Medical Center, Jackson, MS USA; Department of Surgery, University of Mississippi Medical Center, Jackson, MS USA; Department of Internal Medicine, Division of Digestive Diseases, University of Mississippi Medical Center, Jackson, MS USA

Dear Editor:

In the USA, approximately 200 per 100,000 adults are diagnosed with Crohn’s disease (CD). While medical therapy with biologic agents continues to improve patients’ morbidity and mortality, surgical procedures are still required in about half of the patients. Operative management is indicated for patients with refractory disease who develop complications such as bowel obstruction, abscess, fistulas or perforations. There is controversy in the literature on whether side-to-side anastomosis (SSSA) or handsewn end-to-end anastomosis (HEEA) is superior, yet resection of the ileocecal (IC) valve occurs in both [1, 2]. We would like to present a case of a woman with ileal CD who underwent an ileocecal valve sparing ileal resection.

A 58-year-old Caucasian female diagnosed with ileal CD 30 years ago on immunomodulator therapy presented with complaints of abdominal pain consistent with a CD flare. She required hospitalization and initiation of steroid therapy. She was able to taper off of the steroids within 4 weeks. Nine months later, she developed a peri-rectal abscess, which was treated with antibiotics. After resolution of the abscess, she was started on an anti-tumor necrosis factor (anti-TNF) in conjunction with the immunomodulator that she was previously on.

She did well for one year until she was admitted with symptoms of fevers, abdominal pain, diarrhea, and an overall decline in her health. On admission, her labs showed the following: white blood cell count of 10.3 (4.0–10.0 TH/cmm), platelets 452 (150.0–400.0 TH/cmm), hemoglobin 10.1 (12.0–15.0 g/dL), hematocrit 31.2 (36–46 %), c-reactive protein (CRP) 15.7 (0.0–0.5 mg/dL), high sensitivity CRP 182.9 (<3.0 mg/L), erythrocyte sedimentation rate (ESR) 95 (0.0–20.0 mm/h), and basic metabolic panel within normal limits. *Clostridium difficile* was negative. A computed tomography (CT) abdomen and pelvis revealed inflammation in the distal terminal ileum, fistulous tracts extending into the mesentery, irregular thickened walls of the ileum and abscess.

Furthermore, she underwent a colonoscopy, which did not reveal any colonic disease. The terminal ileum was able to be traversed 2 cm with a stricture being noted proximally. Biopsies of the distal ileum revealed small bowel mucosa with focal ulceration, moderate acute and chronic inflammation, and no granulomas, cryptitis, or abscesses. Given the degree of her symptoms and findings, the patient and team decided that a surgical approach would be of benefit.

The patient was taken to the operation room and had a small bowel resection with side-to-side anastomosis of the proximal small bowel and anti-mesenteric wall of the small bowel with closure of the terminal ileum and preservation of the ileocecal valve.

Pathology of the resection revealed mucosal ulceration with granulation tissue and active chronic inflammation with giant cell reaction, submucosal fibrosis consistent with stricture, and focus of ischemic necrosis.

The patient had an uneventful post-operative recovery period and was maintained on an anti-TNF plus imunomodulator. A colonoscopy was performed eight months later and showed normal ileum and colon. Biopsies from the small bowel and colon were normal, suggesting mucosal and clinical remission.

Ileocecal valve preservation is not common surgical practice in CD patients. The IC valve is critical in limiting the reflux of colonic contents into the ileum as well as modulating the rate of small bowel effluent into the colon. Maintaining the integrity of the ileocecal valve while utilizing a SSSA, as done in this patient, may provide many benefits including prevention of small bowel bacterial overgrowth, diarrhea, as well as preserving colonic absorptive surface capacity [3]. Jiang et. al conducted a retrospective study finding that preservation of the IC valve in the pediatric population led to a decreased incidence of diarrhea, intestinal infection and acid-base disturbance [4]. Interestingly, they also determined that those without preservation of the IC valve had significantly decreased serum total bile acid and B12. Additionally, while there is no evidence that a right hemicolectomy increases the risk for colon cancer, it is surmised that increased fecal effluent within the colon may increase dysplasia and hyperplasia. Thus, studies similar to Jiang et. al’s in infants are needed in adults.

In adults, it has been postulated that recurrence and reoperation of CD at or proximal to the ileocolonic anastomosis may occur secondary to the disease process alone. Contributing factors may be increased irritants such as bile acids and increased fecal stream. Guo at el found that there was no difference in endoscopic or symptomatic recurrence of CD utilizing SSSA versus HEEA [3]. Instead of concluding that a particular approach of resecting the ileocecal valve (SSSA vs. HEEA) decreases stenosis, fecal stasis, and colonic reflux, further investigations should focus on the preservation of the ileocecal valve.

In conclusion, adult studies evaluating preservation of the ileocecal valve need to be conducted to elucidate the beneficial effects of prevention of diarrhea, small bowel overgrowth, acid-base imbalances and the theoretical potential of decreasing colonic dysplasia. Our case presents a patient who successfully underwent this surgery and has demonstrated clinical and endoscopic remission eight months after surgery while maintaining on anti-TNF plus immunomodulatory therapy.
